# Maternal Socioeconomic Status and the Risk of Congenital Heart Defects in Offspring: A Meta-Analysis of 33 Studies

**DOI:** 10.1371/journal.pone.0111056

**Published:** 2014-10-27

**Authors:** Di Yu, Yu Feng, Lei Yang, Min Da, Changfeng Fan, Song Wang, Xuming Mo

**Affiliations:** Department of Cardiothoracic Surgery, Nanjing Children's Hospital, Nanjing Medical University, Nanjing, China; University of Birmingham, United Kingdom

## Abstract

**Background:**

We conducted this meta-analysis to address the open question of a possible association between maternal socioeconomic status and congenital heart defects (CHDs).

**Methods:**

We searched MEDLINE and EMBASE from their inception to January 1, 2014 for case-control and cohort studies that assessed the association between maternal socioeconomic status and the risk of CHDs. Study-specific relative risk estimates were polled according to random-effect or fixed-effect models.

**Results:**

From 3343 references, a total of 31 case-control studies and 2 cohort studies were enrolled in this meta-analysis, including more than 50,000 cases. We observed that maternal educational attainment, family income and maternal occupation were negatively associated with an 11% (pooled RR = 1.11, 95% CI: 1.03, 1.21), 5% (pooled RR = 1.05, 95% CI: 1.01, 1.09) and 51% (pooled RR = 1.51, 95% CI: 1.02, 2.24) increased risk of CHDs, respectively. In a subgroup analysis by geographic region, the results were inconsistent for the European region (RR = 1.29, 95% CI: 0.99–1.69) and USA/Canada region (RR = 1.06, 95% CI: 0.97, 1.16) in maternal educational attainment.

**Conclusion:**

In summary, this meta-analysis suggests that a lower degree of maternal socioeconomic status is modestly associated with an increased risk of CHDs. However, further investigations are needed to confirm the association.

## Introduction

Congenital heart defects (CHDs) are the most common group of congenital malformations, affecting almost 1% of live births throughout the world [1]. CHDs represent approximately one-third of all congenital anomalies and are the leading cause of perinatal mortality [2]. Although cardiovascular diagnostics and cardiothoracic surgery have achieved massive breakthroughs over the past century, leading to an increased survival of newborns with CHDs, the etiology of most congenital heart defects is still unknown. Several chromosomal anomalies, certain maternal illnesses, and prenatal exposures to specific therapeutic drugs are recognized risk factors. It is difficult to establish the role of a single factor because the cause of a defect is believed to be multifactorial in many cases, including the combination of environmental teratogens with genetic and chromosomal conditions [3]. A review published in 2007 provided a summary of currently available literature on noninherited risk factors that might alter the risk of CHDs [4]. Moreover, CHDs include several distinct subtypes (e.g., conotruncal defects, left ventricular outflow track defects, and septal defects), and there is a potential for etiologic heterogeneity. Therefore, it is not surprising that studies for categories of CHDs report different or opposite results.

Various approaches to the conceptualization and measurement of socioeconomic status (SES) have been taken, reflecting both different theoretical orientations and the exigencies of conducting studies. In our study, we used the most common measures, indexes, and ecological measures of SES, which is typically characterized by educational attainment, family income level and occupational prestige. According to the International Standard Classification of Occupations (ISCO-08) [5], skill level is used as the criterion for dividing occupations into groups and can be defined as a function of the complexity and range of tasks and duties to be performed in an occupation. This ranking of occupations consists of 4 groups ranging from a low to a high level. Lower SES often has connections with health-damaging lifestyles that result in the development of poor dietary habits and show the influence of behaviors related to physical activity and smoking [6]–[10]. Previous studies have reported that lower SES increases the risk of diabetes mellitus and cardiovascular disease [6], [11]–[14]. Recently, there has been a steep increase in the number of maternal SES studies with CHDs as the primary health outcome, with several studies showing positive associations and others providing null results.

An increasing number of studies to date have focused on the association between maternal SES and CHDs; however, the results have been ambiguous, perhaps because of inadequate sample sizes. Therefore, we conducted a meta-analysis to quantitatively assess the effect of maternal SES on CHDs.

## Materials and Methods

### Literature search

A computerized literature search was conducted by two independent investigators (Yu and Feng) in MEDLINE and EMBASE from their inception to January 1, 2014. We searched relevant studies using the following strategy: (“Socioeconomic Status” OR “Social Class” OR “Middle Class Population” OR “Caste” OR “education” OR “occupation” OR “income”) AND (“abnormalities” OR “birth defects” OR “congenital anomaly” OR “malformations” OR “congenital malformations” OR “congenital heart defect” OR “Heart Abnormality” OR “Malformation of heart” or “CHD”) AND (“maternal” OR “mother” OR “periconceptional” OR “pregnant” OR “gestation”). In addition, we conducted a search with a broader range on environmental teratogens and CHDs and checked the references in relevant retrieved and review articles. In this way, we identified information about other related studies.

### Eligibility Criteria

We selected articles that (1) were original epidemiologic studies (i.e., case–control and cohort), (2) examined the association between maternal SES and CHDs overall or any one of the CHD subtypes in infants, (3) were published in the English language, (4) reported RRs (i.e., risk ratios or odds ratios) and associated 95% confidence intervals (CIs) or had raw data available, and (5) defined CHDs or one of the CHD subtypes as an outcome. Articles that reported results from more than one population were considered separate studies. Non-peer-reviewed articles, experimental animal studies, ecological assessments, correlational studies and mechanistic studies were excluded.

### Data extraction

Data extraction was conducted separately by two reviewers (Yu and Feng) working independently. If differences of opinion arose, these were resolved by discussion between the two. The studies meeting the inclusion criteria were reviewed to retrieve information of interest including study characteristics (i.e., authors, year of publication, geographic region, periods of data collection, study design, sample size, case classification, source of exposure data, and maternal SES (family income, occupational prestige, and education attainment)) and to record reported effect estimates and the associated 95% confidence interval (CI). If effect estimates were not available, raw data were extracted. For original studies that reported risk estimates in association with SES according to more than one measure, each estimate was extracted and its own association with the specific SES then analyzed. The information on the country where the study was conducted was then classified according to the geographical area (USA/Canada, Europe, Asia and Africa) and the country's income level (high-income and middle or low income). The education attainment and family income were evaluated by the lowest vs. the highest reported in the enrolled studies. Information on occupational social class was collected in the evaluated studies and coded according to the International Standard Classification of Occupations (ISCO-08) (unskilled (I), skilled (II), intermediate (III) and professional (IV)).

RR was used as the measure for the summary statistics of associations of maternal SES with CHD risk. To simplify the procedure, RR was used to represent all reported study-specific results from cohort studies and OR from case-control studies. RR estimates and 95% CIs were extracted from each study for CHDs overall and CHD subtypes. To augment the comparability of different SES categories in the studies, the lowest and highest SES categories were compared. We back-calculated the point estimate and 95% CI if the original study did not report the risk estimates in this order. If the original study did not report estimates in the form of RR or OR, we used standard equations to recalculate the risk estimates and 95% CI from the raw data presented in the study.

### Statistical analysis

The strength of the association of maternal SES with CHD risk was evaluated by RR with a 95% CI. We calculated pooled RR and accompanying 95% CI for the lowest vs. the highest categories of both income and education. Occupation included 4 groups, from low to high level, and pooled RRs with 95% CIs were calculated for the first vs. the fourth level, the second vs. the fourth, and the third vs. the fourth.

Cochran *Q* and *I^2^* statistics were used to test for heterogeneity across studies [16]. If there was evidence of heterogeneity (*P*<0.05 or *I^2^*≧50%), the random-effects model was used. This model provided a more appropriate summary effect estimate among heterogeneous study-specific estimates. If the study showed no evidence of heterogeneity, a fixed-effects analysis was used, applying inverse variance weighting to calculate summary RR estimates [17].

Publication bias was assessed by visual inspection of a funnel plot with asymmetry, using both Egger's linear regression [18] and Begg's rank correlation [19] methods. Significant statistical publication bias was defined as a *P* value of <0.05 for the two above-mentioned tests. All statistical analyses were performed with STATA (version 11.0; StataCorp, College Station, Texas, USA).

## Results

### Study characteristics

**Figure 1:**
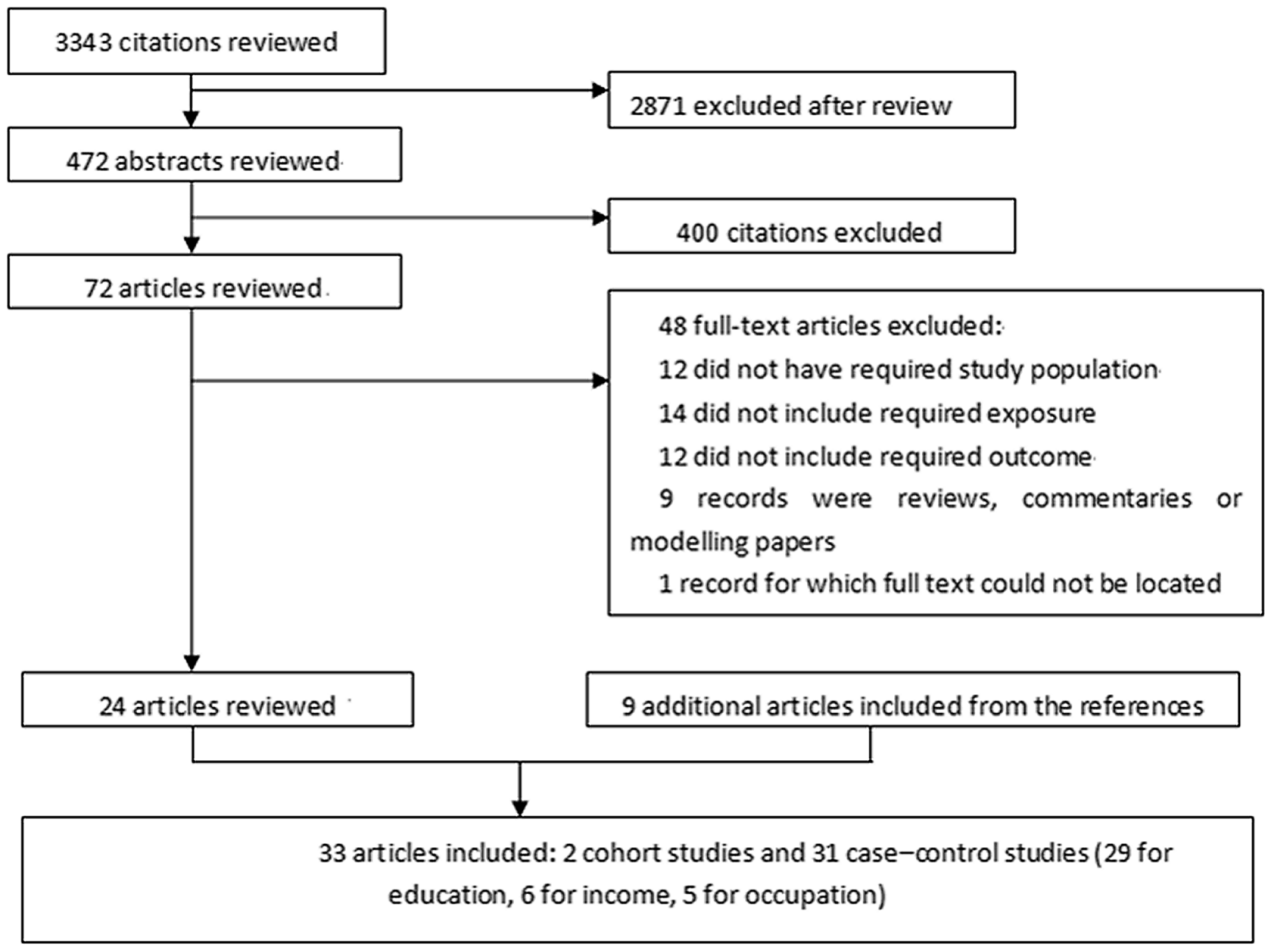
References searched and selection of studies in the meta-analysis.

The search strategy generated 3343 citations, among which 33 were identified in the final analysis for 53,358 incident cases (Figure 1). All of the studies were published from 1989 to 2013. There were 31 case–control studies [15], [20]–[37], [39]–[41], [43]–[48], [52]–[54] and 2 cohort studies [38], [42]. The main characteristics of the included studies are shown in Table 1. In all, 20 studies [16], [23]–[25], [27]–[33], [35], [38], [39], [42]–[44], [46], [52], [54] were conducted in the United States/Canada, 11 in Europe [20]–[22], [34], [36], [37], [40], [41], [45], [47], [53], and 2 in other regions (1 in China and 1 in Egypt) [26], [35]; 29 studies were conducted in high-income countries and 4 in middle or low-income countries. Of the studies examined, 29 investigated the association of educational attainment with CHD risk [16], [20], [21], [23]–[45], [47], [52], [53], 6 examined the association of family income level with CHD risk [23], [24], [29], [31], [39], [54], and 5 examined the association of occupational categories with CHD risk [22], [34], [44], [46], [48].

**Table 1:** Overall characteristics of included studies.

First Author	Year of Publication	Country	Study Design	No. of Cases a	Study Period	Maternal SES Measure
Stoll	1989	Europe	CC	801	1979–1986	Education
Tikkanen	1992	Europe	CC	408	1982–1983	Education
Pradat	1993	Europe	CC	1108	1982–1986	Occupation
Wasserman	1996	USA/Canada	CC	207	1987–1988	Education
Fixler	1998	USA/Canada	CC	89	-	Education; Income
Torfs	1999	USA/Canada	CC	385	1991–1993	Education; Income
Botto	2000	USA/Canada	CC	957	1968–1980	Education
Bassili	2000	Africa	CC	894	1995–1997	Education
Carmichael	2003	USA/Canada	CC	131	1987–1988	Education
Williams	2004	USA/Canada	CC	122	1968–1980	Education
McBride	2005	USA/Canada	CC	476	1991–2001	Education
Batra	2007	USA/Canada	CC	3489	1987–2003	Education
Yang	2008	USA/Canada	CC	397	1997–2000	Education; Income
Grewal	2008	USA/Canada	CC	323	1999–2003	Education
Malik	2008	USA/Canada	CC	3067	1997–2002	Education
Van Driel	2008	Europe	CC	292	2003–2008	Education
Liu	2009	Asia	CC	164	2004–2005	Education
Smedts	2009	Europe	CC	276	2003–2006	Education
Hobbs	2010	USA/Canada	CC	572	1998–2007	Education; Income
Kuciene	2010	Europe	CC	261	1995–2005	Education; Occupation
Long	2010	USA/Canada	CC	1576	1999–2004	Education
Van Beynum	2010	Europe	CC	611	1996–2005	Education
Agha	2011	USA/Canada	Cohort	28302	1994–2007	Education; Income
Alverson	2011	USA/Canada	CC	2525	1981–1989	Education
Karatza	2011	Europe	CC	157	2006–2009	Education
Materna-Kiryluk	2011	Europe	CC	1673	2005–2006	Education
Agopian	2012	USA/Canada	Cohort	563	1999–2008	Education
Lupo	2012	USA/Canada	CC	1907	1997–2002	Education; Occupation
Mateja	2012	USA/Canada	CC	237	1996–2005	Education
Patel	2012	USA/Canada	CC	187	1997–2005	Income
Vereczkey	2012	Europe	CC	302	1980–1996	Occupation
Padula	2013	USA/Canada	CC	822	1997–2006	Education
Vereczkey	2013	Europe	CC	77	1980–1996	Occupation

CC: case–control study; SES: socio-economic status.

a Reported number of cases and control subjects with available exposure information.

### Overall results

**Figure 2:**
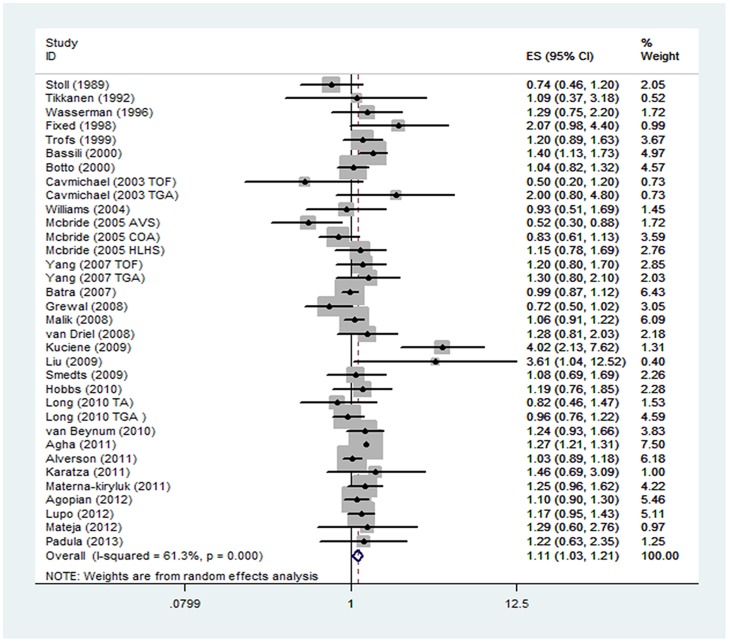
Estimates (95% CIs) of maternal educational attainment (lowest vs. highest category) and congenital heart defect (CHD) risk.

**Figure 3:**
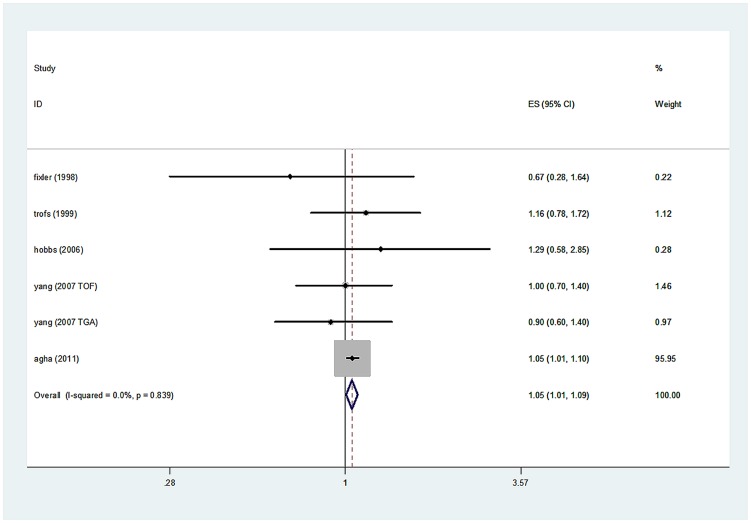
Estimates (95% CIs) of income level (lowest vs. highest category) and congenital heart defect (CHD) risk.

**Figure 4:**
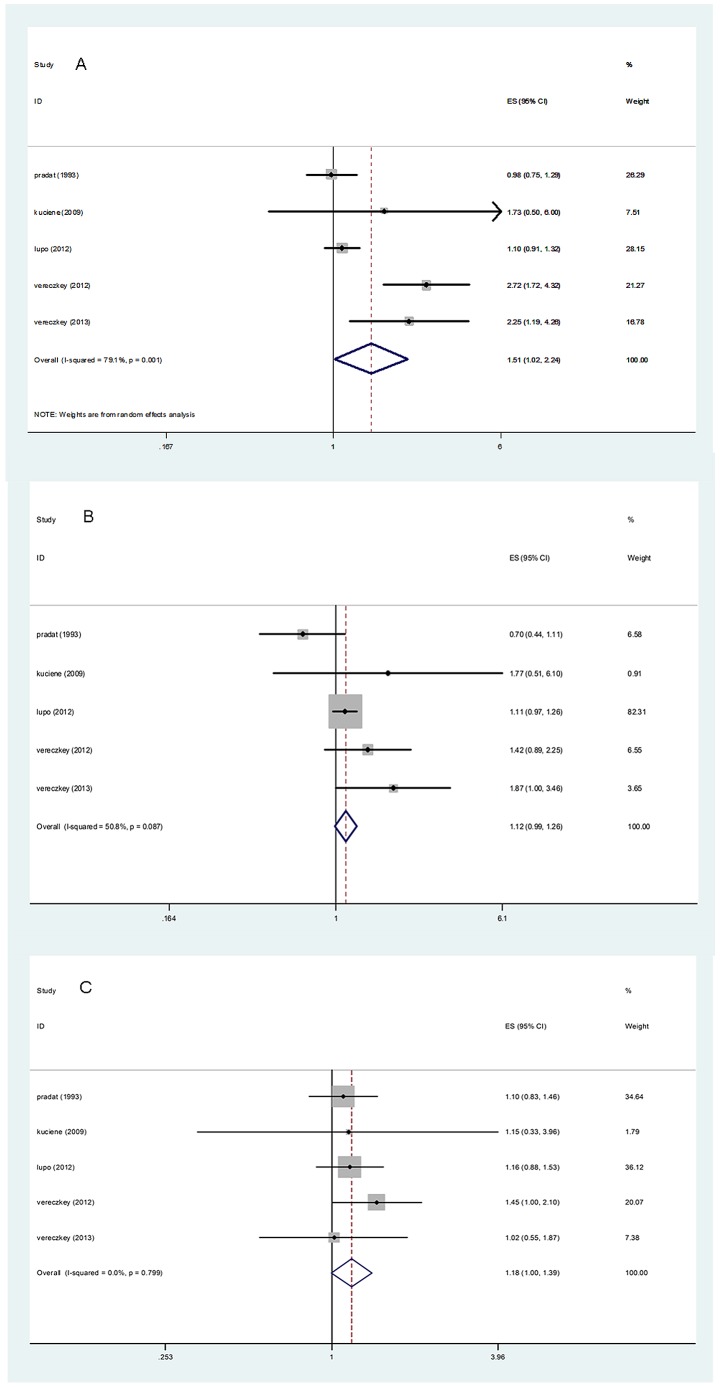
Estimates (95% CIs) of maternal occupational prestige and congenital heart defect (CHD) risk. A: Level I vs. level IV occupation; B: Level II vs. level IV occupation; C: Level III vs. level IV occupation.

The overall results of this meta-analysis provided evidence for a significant increase in the risk of CHDs among the lowest socioeconomic categories for all 3 socioeconomic indicators (figure 2–4). Heterogeneity was observed for education and occupation (p<0.01) (Table 2).

**Table 2:** Pooled estimates for socioeconomic category and incidence of CHDs in series of subgroup analyses.

Subgroup analysis	No. of studies	No. of cases	Education	P ^a^	I^2^(%)	No. of studies	No. of cases	Income	P ^a^	I^2^(%)	No. of studies	No. of cases	Occupation	P ^a^	I^2^(%)
Summary pooled estimate	29	51684	1.11(1.03–1.21)	<0.01	61.3	6	29932	1.05(1.01–1.09)	0.87	0.0	5–	3655	1.51(1.02–2.24)	<0.01	79.1
Country's income group															
High	27	50626	1.10(1.01–1.19)	<0.01	61.3	6	29932	1.05(1.01–1.09)	0.87	0.0	3	3276	1.07(0.92–1.24)	0.59	0.0
Middle or low	2	1058	1.44(1.17–1.77)	0.14	53.8	0	-	-	-	-	2	379	2.55(1.76–3.70)	0.64	0.0
Geographical region															
USA/Canada	19	46147	1.06(0.97–1.16)	<0.01	62.6	6	29932	1.05(1.01–1.09)	0.87	0.0	1	1907	1.10(0.91–1.32)	-	-
Europe	8	4479	1.29(0.99–1.69)	0.01	61.4	0	-	-	-	-	4	1748	1.76(0.93–3.32)	<0.01	82.2
Asia	1	164	3.61(1.04–12.52)	-	-	0	-	-	-	-	0	-	-	-	-
Africa	1	894	1.40(1.13–1.73)	-	-	0	-	-	-	-	0	-	-	-	-
Sample size															
≤1000	9	2273	1.26(1.05–1.52)	0.33	12.7	3	1046	1.12(0.86–1.48)	0.81	0.0	1	302	2.73(1.72–4.32)	-	-
>1000	20	49411	1.09(0.99–1.19)	<0.01	69.2	3	28886	1.05(1.00–1.09)	0.86	0.0	4	3353	1.11(0.96–1.29)	0.11	50.2
Publication period															
Before 2004	8	3872	1.19(1.05–1.35)	0.07	44.2	2	89	1.06(0.74–1.52)	0.27	19.0	1	1108	0.98(0.75–1.29)	-	-
2004 or after	21	47812	1.10(1.00–1.20)	<0.01	66.2	3	29843	1.05(1.01–1.09)	0.87	0.0	4	2547	1.80(1.01–3.24)	<0.01	81.3
Design															
Case-control	27	22819	1.10(1.01–1.20)	<0.01	47.8	5	1630	1.02(0.86–1.22)	0.79	0.0	5	3655	1.51(1.02–2.24)	<0.01	79.1
Cohort	2	28865	1.26(1.21–1.31)	0.13	55.4	1	28302	1.05(1.01–1.10)	-	-	0	-	-	-	-

^a^ P value for heterogeneity

### Association of SES categories with CHD risk

**Figure 5:**
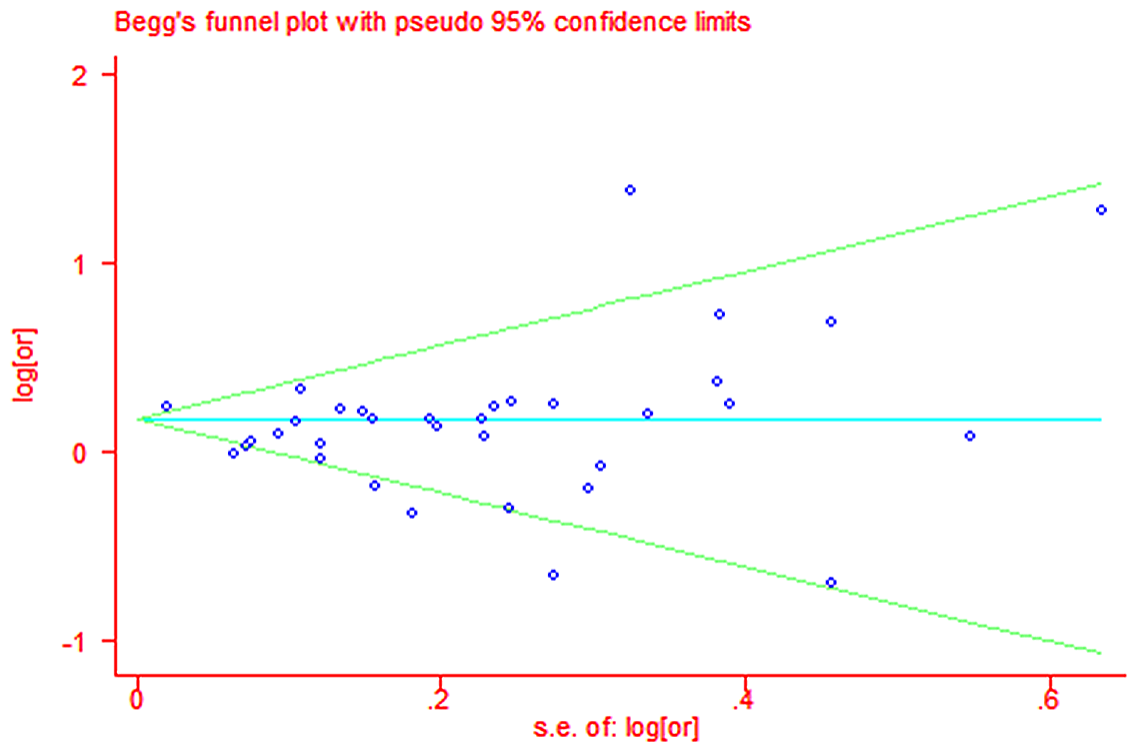
Begg's test of studies for maternal educational attainment and congenital heart defects (CHD).

A total of 29 studies evaluated the association between maternal educational attainment and CHDs as a group (Table 2). We found that decreasing maternal educational attainment was associated with an 11% increase in the risk of CHDs (RR = 1.11, 95% CI: 1.03, 1.21) (Figure 2). Statistically significant heterogeneity was detected (Q = 85.32, *P*<0.001, *I^2^* = 61.3%), with no publication bias (Begg's test: P = 0.68, Egger's test: P = 0.14) (Figure 5). After stratification by the countries' income group, a 10% increment was found among high-income countries (RR = 1.10, 95% CI: 1.01, 1.19); additionally, a significant increment (44%) was found among middle- or low-income countries in education (RR = 1.44, 95% CI: 1.17, 1.77). Furthermore, in a subgroup analysis by geographic region, null results were found for European (RR = 1.29, 95% CI: 0.99–1.69) and North American studies (RR = 1.06, 95% CI: 0.97, 1.16). If the analysis was limited to cohort studies (RR = 1.26, 95% CI: 1.21, 1.31) and sample sizes under 1000 (RR = 1.26, 95% CI: 1.05, 1.52), the results were generally consistent with the overall summary measure. Moreover, a significant association was observed in case-control studies (RR = 1.10, 95% CI: 1.01, 1.20), whereas no significant association was observed for sample sizes greater than 1000 (RR = 1.09, 95% CI: 0.99, 1.19). An increased incidence of CHD was observed if studies on family income were pooled (RR = 1.05, 95% CI: 1.01, 1.09) (Table 2), with no heterogeneity (Q = 2.49, P = 0.87, *I^2^* = 0.0%) or publication bias (Egger's test: *P* = 0.475) (Figure 3). For the influence of occupation, Figure 4 shows the relationship between SES, categorized by occupation in classes one to four, and CHDs. In most of the studies reviewed, the risk of CHDs was higher in the lowest classes and affected the entire SES spectrum: the first vs. the fourth (RR = 1.51, 95% CI: 1.09–2.24), the second vs. the fourth (RR = 1.12, 95% CI: 1.00, 1.26) and the third vs. the fourth (RR = 1.18, 95% CI: 1.00, 1.39). In high-income countries, lower maternal occupational prestige was associated with a 7% increased risk of CHDs (for the first level vs. the fourth level, RR = 1.07, 95% CI: 0.92, 1.24); additionally, a significant increment (155%) was found for middle- or low-income countries (RR = 2.55, 95% CI: 1.76, 3.70).

## Discussion

The purpose of the current study was to investigate the association between SES and CHDs. SES is customarily determined by educational achievement, family income and occupational prestige. To examine this association, we conducted a meta-analysis including 29, 6 and 5 studies of maternal education, family income and maternal occupation, respectively. Our findings indicated an increased incidence of CHDs among the lowest SES classifications in maternal education (11%), family income (5%) and maternal occupation (51%) compared with the highest classification of the corresponding SES. Moreover, for education stratified by the country's income group, a 10% increment was found among high-income countries. Furthermore, a significant increment (44%) was found among middle- or low-income countries. The results were inconsistent with the findings of the subgroup analysis of geographic regions, most likely indicating that in addition to individual SES, regional SES differences, especially between the developed countries and developing countries, may also have an influence on the risk of CHDs. Many factors could explain this finding. First, poor education is always accompanied by smoking [49] and diabetes mellitus [50], which have both been shown to be associated with an increased risk of CHDs [10], [51]. Moreover, education has attracted much attention in developed countries, where everyone can receive a good education. Thus, there are no significant differences among individuals, and this uniformity may lead directly to the results. However, the opposite pattern occurs in developing countries. The unequal distribution of educational resources contributes to the obvious individual differences. However, the number of studies including a subgroup analysis of middle- or low-income countries is limited; for this reason, more studies need to be included in the meta-analysis to further confirm our findings. In regard to family income and occupational prestige, our results showed that the lowest income category and occupation levels increased the risk of CHDs compared with the highest. This result was consistent with the conclusions for education. Moreover, the percentage increment in risk decreased as the occupational level increased (Level I vs. Level IV RR = 1.51, 95% CI: 1.09–2.24; Level II vs. Level IV RR = 1.12, 95% CI: 1.00, 1.26; Level III vs. Level IV RR = 1.18, 95% CI: 1.00, 1.39). However, as a limited number of studies were included, the results need further confirmation. Moreover, note that case-control studies are always accompanied by selection and information biases. The studies that we examined are almost all case-control studies. Accordingly, the estimates of the relationship of SES in education to CHD risk based on case-control studies (RR = 1.10, 95% CI: 1.01, 1.20) are consistent with the conclusion drawn from the pooled analysis.

Several limitations of our study should be considered. First, a total of 31 case-control studies and 2 cohort studies were recruited in our meta-analysis, and we extracted our raw data principally from case-control studies, which are susceptible to selection and information biases. Therefore, our results cannot be viewed as an inevitable relationship, and further investigations including more high-quality studies are needed. Second, our meta-analysis was limited to studies published in English, but no evidence of publication bias was found, whereas heterogeneity exists in the component studies. This finding may reflect the differences among study designs and study populations as well as other unknown factors associated with the included studies. Moreover, as our study was limited to English-language publications, our results may have been affected by the lack of data from studies published in other languages, especially in the middle- or low-income countries, where SES may be associated with CHDs. Therefore, any general conclusions must be considered carefully. The third limitation of our meta-analysis is the possible differences in the classification and definition of SES among the examined studies. Countries' overall economic and educational levels have a significant impact on the SES categories of maternal educational achievement, family income and maternal occupational prestige. Meanwhile, family income cannot strictly be reported as a solely maternal characteristic and we used it to instead of maternal income, which would bring confounding factors. Finally, our small sample size for family income and maternal occupation levels may have been underpowered to detect any influence of SES on the risk of CHD. Additionally, lacking a large set of data, we did not conduct a subgroup analysis of CHD subtypes.

However, our study offers several important strengths. Due to the difficulty of evaluating the socioeconomic level and the lack of sufficient available literature, no meta-analysis had previously been performed to investigate the association of maternal SES with the risk of CHDs. Therefore, to our knowledge, this is the first meta-analysis to report an association between maternal SES and CHDs, including more than 50,000 cases. Moreover, our literature search was conducted on multiple databases, and the references in the relevant retrieved and review articles were fully scrutinized to obtain the missing data. Moreover, both Egger's linear regression and Begg's rank correlation tests showed no significant publication bias.

In summary, this study provides evidence of an association between low SES and an increased risk of CHDs, including maternal educational attainment, family income and maternal occupational prestige, whereas no clear relationship was found between socioeconomic status and CHDs in developed countries. Our findings could make public health policy focus more strongly on at-risk populations and could be used in the development of population-based prevention strategies to reduce the incidence and burden of CHDs, particularly for the regions with a lower level of economic development. Moreover, as previous studies have found a correlation between educational level and CHD risk factors [50], [51], maternal education attainment appears to be the main target for preventing the development of CHDs.

## Supporting Information

Checklist S1 **PRISMA checklist.**
(DOC)
